# Balancing beauty and science: a review of facial implant materials in craniofacial surgery

**DOI:** 10.3389/fsurg.2024.1348140

**Published:** 2024-01-24

**Authors:** Martin Kauke-Navarro, Leonard Knoedler, Samuel Knoedler, Can Deniz, Lars Stucki, Ali-Farid Safi

**Affiliations:** ^1^ Department of Surgery, Division of Plastic and Reconstructive Surgery, Yale New Haven Hospital, Yale School of Medicine, New Haven, CT, United States; ^2^ Craniologicum, Center for Craniomaxillofacial Surgery, Bern, Switzerland; ^3^ Faculty of Medicine and Dentistry, Danube Private University, Krems, Austria; ^4^ Medical Faculty, University of Bern, Bern, Switzerland

**Keywords:** implant, face, zygoma, reconstruction, facial augmentation

## Abstract

Facial reconstruction and augmentation, integral in facial plastic surgery, address defects related to trauma, tumors infections, and congenital skeletal deficiencies. Aesthetic considerations, including age-related facial changes, involve volume loss and diminished projection, often associated with predictable changes in the facial skeleton. Autologous, allogeneic, and alloplastic implants are used to address these concerns. Autologous materials such as bone, cartilage, and fat, while longstanding options, have limitations, including unpredictability and resorption rates. Alloplastic materials, including metals, polymers, and ceramics, offer alternatives. Metals like titanium are biocompatible and used primarily in fracture fixation. Polymers, such as silicone and polyethylene, are widely used, with silicone presenting migration, bony resorption, and visibility issues. Polyethylene, particularly porous polyethylene (MedPor), was reported to have one of the lowest infection rates while it becomes incorporated into the host. Polyether-ether-ketone (PEEK) exhibits mechanical strength and compatibility with imaging modalities, with custom PEEK implants providing stable results. Acrylic materials, like poly-methylmethacrylate (PMMA), offer strength and is thus mostly used in the case of cranioplasty. Bioceramics, notably hydroxyapatite (HaP), offer osteoconductive and inductive properties, and HaP granules demonstrate stable volume retention in facial aesthetic augmentation. Combining HaP with other materials, such as PLA, may enhance mechanical stability. 3D bioprinting with HaP-based bioinks presents a promising avenue for customizable and biocompatible implants. In conclusion, various materials have been used for craniofacial augmentation, but none have definitively demonstrated superiority. Larger randomized controlled trials are essential to evaluate short- and long-term complications comprehensively, potentially revolutionizing facial balancing surgery.

## Introduction

Restoration of facial form and function after trauma, tumor, infection or in the setting of congenital skeletal deficiencies is an integral part of a facial plastic surgeons practice. Aesthetic considerations such as treatment of issues related to the aging face which includes volume loss, imbalanced facial features and definition can be addressed with autologous, allogeneic or alloplastic implant augmentation. Changes of the aging face are often related to predictable changes of the facial skeleton, with loss of projection and volume in particular in the anterior parts of maxilla and mandible (1). Hence, for aesthetic considerations facial implants are most commonly used to augment the malar eminences, chin, as well as the mandibular angle/ramus (2).

Facial implantology uses various types of implants such as metals, polymers [e.g., polysiloxane (silicone), polyethylene (porous polyethylene = medpor), Polytetrafluoroethyelene (Gore-Tex), Methylmethacrylate (MMA)] and ceramics (e.g., hydroxypaptite) (3).

The most commonly used materials are microporous high-density polyethyelene - (Medpor) and silicone implants (2, 4). Although autologous options have long represented the gold-standard for facial implantology (“augment like with like tissues”), several disadvantages are inherent to these options (4). Disadvantages include added donor site morbidity of bone or soft tissue harvest, increased operative times and often unfavorable but predictable attrition rates leading to less stable outcomes of facial augmentation (4).

Although a handful of reviews discuss complications and try to define the short and long-term risk, there is no study condensing the current body of literature in a comprehensive way with regards to different implant types, indications, and related complications. The herein presented work aims to summarize the risk profile of currently available facial implant materials while discussing future developments in this exciting field.

### Autologous tissues for facial augmentation

#### Bone

Autologous bone onlay grafts were found to be largely unpredictable in terms of stability of the augmentation result due to bony resorption (5). Most commonly, such bone grafts are harvested from the calvarium or iliac crest. In comparison, calvarial onlay grafts were found to be more form stable when compared to bone harvested from the iliac crest, likely due to the higher relative volume of cortical bone that is naturally more resistant to osteoclastic resorption (4, 6). Due to the unpredictable volume retention rates, aesthetic facial augmentation is typically done with more reliable alloplastic materials that are less prone to resorption and loss of structure over time (4).

#### Cartilage

Autogenous (e.g., harvested from the ribs, ear, nasal septal grafts) and lyophilized (freeze-dried) allogeneic bank cartilage can be used for facial augmentation (7). Lyophilized allogenic cartilage is freeze-dried and taken from organ donors with good acceptance rates. This type of graft has been used extensively in the reconstruction and augmentation of the craniofacial skeleton including specifically nasal reconstruction (e.g., saddle deformity), facial asymmetry and contour reconstruction, and other bony defects (8). The infection rate was reported to be as low as 2.6%, however with up to 20% resorption (7). Additionally, cartilage grafts tend to warp and are thus not suitable for areas of augmentation that require strict form stability. In rhinoplasty revision cases when autologous septal cartilage for grafting may be scarce, fresh frozen allogeneic costal cartilage grafts (FFCC) from the musculoskeletal Transplant Foundation (MTF) were shown to be a safe option in primary and revision rhinoplasty used as columellar strut, septal extension, alar contour, dorsal onlay and lateral nasal wall grafts. Overall rate of complications were reported to be low with infection up to 2.7% (226 patients w 54% revision cases, 12 months average follow up) and in another study (282 cases w 82% revision cases) none of the patients had signs of cartilage warping, unwanted resorption, infections or displacement at 20 months average follow up (9, 10).

##### Lyophilized cartilage

In our own experience, lyophilized cartilage's biocompatibility is a foremost attribute, facilitating integration with the host tissue without eliciting notable adverse immunological responses. The preservation technique, which involves freezing and dehydration, retains the native extracellular matrix structure of the cartilage. This ensures that the graft, once rehydrated and implanted, can readily assimilate with surrounding tissues, paving the way for optimal healing and integration.

A notable characteristic of lyophilized cartilage is its surgical adaptability. The material can be sculpted intraoperatively, allowing for tailored grafting solutions specific to the patient's anatomical requirements, thus optimizing both aesthetic and functional outcomes. Therefore, it is our surgeons experience, that this material is for example a very beneficial material for zygomatic bone augmentation for patients with central and lateral midface hypoplasia (i.e., after large advancements of the upper jaw in the LeFort-I level or of the midface in the LeFort-II level).

Furthermore, the use of lyophilized cartilage diminishes donor site morbidity concerns, as it obviates the need for autologous cartilage harvest, a procedure which can introduce additional complications and patient discomfort.

In the realm of tissue preservation, the lyophilization process effectively reduces the graft's antigenicity while preserving its biomechanical and biochemical properties. This ensures that the grafted material incites minimal inflammatory or foreign body reactions in the recipient site.

Generally, we use lyophilized cartilage for zygomatic bone augmentation, especially if the forward displacement of the upper jaw is greater and harmonization of the midface is necessary as a result. In this case, we can cut lyophilized cartilage to size intraoperatively and insert it into a tissue cavity prepared to accommodate the desired volume.

##### Brief description of our protocol for lyocartilage augmentation of the zygoma

The procedure begins with infiltration of the oral vestibulum using 1% lidocaine with epinephrine. An incision is made in the vestibular mucosa (bilaterally in isolated augmentation cases) and dissection is carried down to the zygomaticoalveolar crest. A subperiosteal dissection is performed, creating a pocket that accommodates the desired cartilage volume and patient-specific augmentation needs. It is important to not overdissect the pocket. Based on preoperative planning and discussion with the patient, the lyocartilage is cut into the desired shape intraoperatively, and consecutively introduced into the subperiosteal pocket. After confirming the desired effect on the facial contour, the cartilage is fixed to the overlying soft tissues using absorbable sutures (3–0 vicryl). A layered closure is then performed.

#### Autologous fat

Autologous fat transfer (donor sites e.g., thigh, abdomen) is a frequently used adjunct in aesthetic facial plastic surgery for balancing of facial features. However, fat grafting cannot replace skeletal augmentation as only skeletal augmentation will be able to achieve projection and definition of facial contour. Augmentation of the soft tissue envelope alone will rather lead to loos of definition (4). The technique of autologous fat transfer in the setting of facial contour augmentation was discussed and described in detail by Kaufman et al. in 2007 (11). Fat retention rates were studied in breast fat grafting and demonstrated volume retention rates of 50%–80% (12).

### Alloplastic materials

The main groups of alloplastic materials for facial implantology include metals such as titanium, polymers (e.g., polysiloxane, polyethylene, polytetrafluoroethylene, proplast, methylmethacrylate) and ceramics (e.g., hydroxyapatite) (3, 13). These materials have different properties and use cases depending on the function a certain implant is desired to have (e.g., load-bearing, augmentation, protection). A summary of different materials and their advantages/disadvantages are provided in Table 1 (33).

**Table 1:** Overview of use cases, advantages, disadvantages and cost for commonly used materials in craniofacial reconstruction and augmentation.

Material	Use	Advantages	Disadvantages
Allogeneic cartilage (14–17)	Primary and revision rhinoplasty, ear reconstruction, structural airway support in facial paralysis patients	High biocompatibility Easy intraoperative adaptability	Warping, non-load bearing, variable resorption/integration, immunogenic potential
Titanium (18–20)	Cranioplasty, orbital reconstruction, titanium plating systems, mandibular reconstruction	High mechanical strength, corrosion-resistant, low weight, imaging compatibility, osseointegrates	Thermal sensitivity stress shielding, no osteoconduction, allergic potential
Silicone (21, 22)	Occipital/temporal implants, rhinoplasty	Easy removal, flexibility, low allergic risk	Bony resorption, lack of integration and formation of capsule, no osteoconduction, imaging artifacts
(porous) Polyethylene (2)	Microtia reconstruction, genioplasty, malarplasty	Incorporation as it allows tissue ingrowth, imaging compatibility	Difficult to remove due to incorporation and porosity, thermal sensitivity
Polyetheretherketone (PEEK) (23, 24)	Cranioplasty, facial implant augmentation, orbital reconstruction	High chemical stability, low toxicity, low thermal reactivity, high mechanical strength, imaging compatibility, intraoperative modeling	Low bioreactivity (poor interfacial adhesion), higher incidence of infections
Polymethylmethacrylate PMMA (25–28)	Cranioplasty, rhinoplasty, facial implants	Radiolucent, easy to mold prior to polymerization, cost-effective	Hardens in exothermic reaction, risk of tissue injury, difficult to re-shape after polymerization, risk of plate fracture and infections, lack of osseointegration
Hydroxyapatite (29–32)	Cranioplasty, facial implantology, orbital reconstruction, rhinoplasty	Clinically used for >20 years, biocompatible, osteoconductive and becomes osseointegrated, volume stability	Pure HaP can brittle, processing and preparation costs

Both the material used, and location seem to play a role in the relative frequency of complications. The highest infection rates were seen in patients who receive implants for malar augmentation (2.67%) followed by frontal bone region (2.5%) and nasal implants (1.75%), overall likely related to the exposure of the implant to mucosal surfaces (34). Complication rates for specific implants are discussed below.

#### Metals

Titanium is the standard material used for craniofacial plating systems due to its inherently high mechanical strength and corrosion resistance (35). Titanium is highly biocompatible and not ferromagnetic, demonstrates excellent durability and low weight, thus making it suitable for MRI imaging and has low artifact on CT. Titanium is very stable and rigid (with an elastic modulus higher than native bone), making it ideal for craniofacial fracture fixation, plating and less so for purposes of augmentation, especially in the aesthetic setting (35, 36). The higher mechanical strength and resistance to deforming forces can lead to a “stress shielding effect” on the adjacent bone, leading to loss in structure and strength of the surrounding native bone (37). This effect can lead to unwanted loosening of the implant (38). The use of metal fixation in craniofacial surgery has widely been studied. Complication rates vary depending on the location with plates used for mandibular reconstruction showing the highest incidence of overall complications (14% infection rate, 20% plate removal required) (3). Despite its excellent biocompatibility, corrosion resistance and strength, titanium can be visible through thin areas of skin under certain circumstances, particularly in the periorbital region or other areas with thin skin. This can lead to undesirable aesthetic results.

Other well-known issues related to titanium implants and plates are thermal sensitivity/intolerance (due to higher thermal conductivity than surrounding tissues which can lead to a unpleasant feeling of cold in the area) and discomfort which often requires removal (39).

#### Polymers

Polymers are large organic macromolecules that are composed of a high number of equivalent sub-molecules. A naturally occurring polymer is collagen (tropocollagen polymer). Facial implantology typically uses synthetic polymers such as medical grade polysiloxane (Silicone) and polyethylene (34).

##### Silicone

Silicone implants are ubiquitously used with good biocompatibility and are one of the most commonly used material for facial implantology (36). Silicone polymers can behave differently based on their degree of total number of chains and complexity of crosslinkage (degree of polymerization and cross-linkage). Vulcanization is the methods that allows creation of longer-chain highly crosslinked silicone polymers that increases the viscosity. Silicone can thus be prepared as a solid, gel or be used in liquid formulation depending on the chemical properties. The material is typically incapsulated by a fibrous membrane over time and is not incorporated into the host. This makes removal simple, however can lead to seroma formation, unwanted shift of the implant, extrusion, poor aesthetic results and thus may require removal (36). These features, however, make silicone implants ideal for breast reconstruction.

Common complications related to the use of silicone for facial augmentation is the possibility of migration, bony resorption, visibility of soft tissue capsule. In a review, silicone for augmentation of malar, chin and mandible contour was associated with a 5% complication rate (2.2% infection) and bone resorption occurred in 55% of patients (2). Reoperation with removal was necessary in about 4.1% of patients. In a larger meta-analysis including 443 patients (all facial areas), infection rate was low at 1.6% with overall low complication rates. Poor cosmetic outcome was the lowest (compared to other commonly used materials such as Medpor) at 2.7% (34). Rubin and Yaremchuk reported that silicone implants had one of the highest rates of removal due to implant related complications (11.7% compared to 0.5% of porous polyethylene and 1.3% of hydroxyapatite) (4).

##### Polyethylene

Polyethylene is another common polymer used in facial implantology. Based on composition, polyethylene polymers can have a wide range of consistencies and mechanical durability.

In contrast to silicone implants, polyethylene implants such as MedPor are porous implants (100–150 µm pore size) that support ingrowth of tissue by the host and thus less likely to be incapsulated but rather incorporated (40). Porous polyethylene implants can be modified intraoperatively to achieve the desired results for a patient and are typically screw fixated. Removal of porous implants such as MedPor is much more difficult compared to silicone implants due to the ingrowth of the hosts connective tissue. In case of MedPor for microtia repair this is a desired feature (41). Medpor complications for similar location as silicone implants (malar, chin, mandible) was only 0.7% infections, 5% need for removal or reoperation and a rate of 3.9% for undesired prominence (vs. only 1.4% for silicone implants). In a larger meta-analysis including all areas of the face, infection rate was found to be about 1.2%. Poor cosmetic outcome was noted in about 5% (34).

##### Polyether-ether-ketone (PEEK)

Polyether-ether-ketone (PEEK) is another polymer that is commonly employed for cranial vault reconstruction, spine surgery and facial augmentation due to its inherent high mechanical strength, low interference with CT/MRI imaging modalities (radiographically translucent) and excellent biocompatibility. Custom made PEEK implants have been used with high success rates in cranial vault reconstruction, with overall low complication rates (42). In small sample cohort study, it was shown that custom made PEEK onlay implants can become osseointegrated over time (bone formation around the implant) (24). Studies indicate that titanium covered PEEK implants have even better osseointegrative properties in preclinical studies (43). In another study, custom made PEEK implants were used for augmentation of various defects. The reported infection rate was 8% which is slightly higher compared to other materials as described above (44). This is likely related to the tendency of PEEK implants to favor biofilm formation/bacterial adhesion compared to other implant materials (38). In a recent study, Sarfraz et al. compared the behavior of different bacterial strains (S. mutans, aureus, E. faecalis, E. coli) on Titanium and PEEK implants with either saliva contamination or without (38). The authors showed that (a) saliva coating of the implant material led to improved attachment of most bacteria to the material and (b) that PEEK allowed for better adhesion than titanium (38).

In our practice, PEEK implants coated with hydroxyapatite (intended to improve osseointegration and thus positional stability of the implant) are used with good success. Hydroxyapatite coating of PEEK implants was shown to have improved osseointegration and fixation of the implants (45). Other coating strategies include bioglass (calcium sodium phosphosilicate) and β-tricalcium phosphate (46).

PEEK's radiolucency stands out as a distinct advantage, ensuring transparency in radiological images. This permits a clear postoperative assessment, unmarred by the artifacts commonly associated with metallic implants. In the realm of biomechanics, PEEK's modulus of elasticity mirrors that of cortical bone. This similarity minimizes stress shielding between the implant and surrounding bone tissue, potentially fostering improved osseointegration and long-term stability.

A salient benefit of PEEK is its adaptability during surgery. Unlike many implant materials that are rigid in their form, PEEK allows for intraoperative modifications. This malleability ensures a precise fit tailored to the patient's specific anatomical nuances, paving the way for enhanced aesthetic and functional outcomes. From a tissue compatibility standpoint, PEEK implants have shown commendable biocompatibility in my practice, manifesting minimal inflammatory or foreign body reactions in surrounding tissue.

In conclusion, PEEK, with its unique properties and bolstered by clinical evidence, emerges as an excellent choice for craniofacial surgical applications. Its advantages in imaging, biomechanical compatibility, intraoperative adaptability, and tissue tolerance underscore its prominence as a preferred material in this domain.

##### Acrylic materials

An example of an acrylic material is poly-methylmethacrylate (PMMA) and it has been used in the setting of many craniofacial defect repairs including orbit, malar eminence and skull defects (4, 36). PMMA is radiolucent and does not interfere with standard imaging modalities. Similar to silicone, PPMA is non-absorbable and gets encapsulated and not incorporated by the host. The material is characterized by initially easy pliability after mixing the monomeric powder and liquid polymer. Over time the material hardens in an exothermic reaction into the desired shape and becomes a material with high mechanical resilience (40). PMMA has extensively been used for cranioplasty.

In a meta-analysis of facial implant materials, PMMA was found to have the highest relative rate of hematoma formation after implantation (6%). Infection was noted in approximately 3% of cases (34). However, due to difficulties re-shaping this material, the risk of plate fractures and infections as well as the lack of osseointegration, this material is not typically used for midfacial augmentation or reconstruction, especially in load bearing areas. Of note, for soft tissue augmentation substances such as Artecoll (PMMA-Microspheres) exist and can be used for correction of for example facial folds, augmentation of lip, chin and malar eminence (47).

#### Bioceramics

Hydroxyapatite (HaP, ceramic composed of calcium and phosphate) is the main inorganic phase of human bone and thus synthetic HaP has a unique role in the field of bone tissue engineering due to its inherently high osteo-conductivity and inductivity (48). HaP based implants were shown to have superior properties with regards to promotion of cellular attachment and integration compared to other alloplastic implants (49). Thus, HaP becomes incorporated into the recipient and is less likely to require removal when compared to silicone/titanium implants. HaP is commercially available in both block and granular form. For facial augmentation, the granular form is often layered in a subperiosteal plane without the need for screw fixation. The use of hydroxyapatite granules seems to be volume stable with a study demonstrating volume stability of 99.7% at 2 year follow up (50).

In a recent study, the outcome of using porous HaP granules for facial aesthetic augmentation was evaluated. In this study, the authors describe their technique of subperiosteal HaP onlay grafting of zygoma, anterior maxilla and mandible. Over a follow up period of 5 years, HaP was removed from 17 patients (due to balance imperfections related to the augmentation, cohort of >500 patients) and the composition was studied. The authors found that the HaP granules were essential encased by host collagen and then gradually replaced by neo-bone including osteoblasts and osteocytes, confirming the excellent bio-acceptance of HaP for facial augmentation (51).

HaP-based scaffolds have been used as described above, however pure HaP scaffolds are generally brittle. To overcome some of the stability concerns, HaP was combined with other substances for example PLA (polylactic acid) to increase mechanical strength and form stability (52, 53). In a recent study, it was indeed demonstrated that the mechanical strength of such constructs (PLA/HA) are comparable to trabecular bone (53).

**Figure 1:**
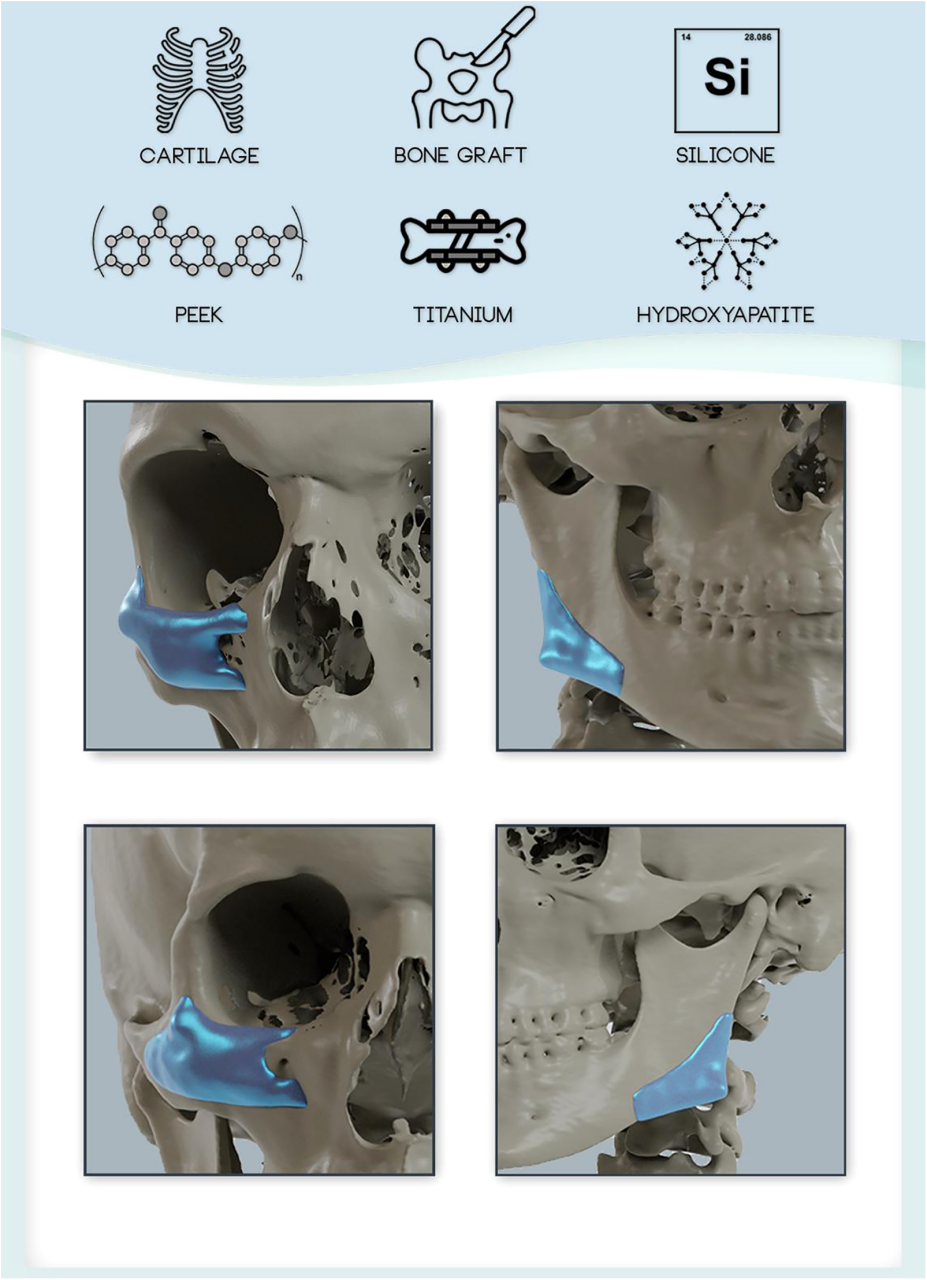
Overview of available implant materials for craniofacial reconstruction and augmentation. Autologous and allogeneic tissues (e.g., cartilage, bone) and alloplastic materials (e.g., silicone, polymers such as PEEK, Titanium, and ceramics (e.g., Hydroxyapatite) are available for reconstruction and augmentation of the craniofacial skeleton. Custom made implants can be tailored to the patient's individual needs and anatomy to achieve an optimized fit (shown at the example of zygoma/infraorbital and mandibular angle implant designs).

In this context, 3D bioprinting has emerged as a possible solution for a more customized approach to bony defect reconstruction and facial balancing surgery using HaP based bioinks. Recent advances have attempted to combine bioinks (printable biomaterials) such as GelMA (gelatin with methacryloyl side groups) with hydroxyapatite. The combination bioinks with HaP was demonstrated to improve stability of the bioink and promote osteoblastic differentiation and mineralization in a preclinical study (54). In the future, customized 3D bioprinted constructs may revolutionize facial balancing surgery by providing excellent biocompatible, form stable and customizable implants (Figure 1).

## Conclusion

Many implants exist for augmentation of the craniofacial skeleton. Due to the unreliable nature of autologous grafts as well as added operative time and donor site morbidity, alloplastic materials are most commonly used for facial contour reconstruction and augmentation. Not one of the materials has demonstrated clear short- and long-term superiority in the literature. However, some materials have distinct disadvantages. Materials such as Tiatanium, HaP and PEEK have been used successfully for custom creation of patient specific implants. In the future, larger ideally randomized controlled trials are performed to analyze in depth the short- and long-term complications of such implants.
